# Genomics to understand the global landscape of linezolid resistance in Enterococcus faecium and Enterococcus faecalis

**DOI:** 10.1099/mgen.0.001432

**Published:** 2025-06-18

**Authors:** Jia Qi Beh, Diane S. Daniel, Louise M. Judd, Ryan R. Wick, Peter Kelley, Katie M. Cronin, Norelle L. Sherry, Benjamin P. Howden, Christopher H. Connor, Jessica R. Webb

**Affiliations:** 1 Department of Microbiology and Immunology at the Peter Doherty Institute for Infection and Immunity, The University of Melbourne, Melbourne, Australia; 2 Microbiological Diagnostic Unit Public Health Laboratory, Department of Microbiology and Immunology at the Peter Doherty Institute for Infection & Immunity, The University of Melbourne, Melbourne, Victoria, Australia; 3 Centre for Pathogen Genomics, University of Melbourne, Melbourne, Victoria, Australia; 4 Department of Infectious Diseases, Peninsula Health, Frankston, Victoria, Australia; 5 Department of Microbiology, Eastern Health, Box Hill, Victoria, Australia; 6 Department of Microbiology, Royal Melbourne Hospital, Melbourne, Australia; 7 Department of Infectious Diseases & Immunology, Austin Health, Heidelberg, Victoria, Australia; 8 School of Biological Sciences, The University of Adelaide, Adelaide, South Australia, Australia

**Keywords:** antimicrobial resistance, *Enterococcus faecalis*, *Enterococcus faecium*, epidemiology, genomics, linezolid, public health

## Abstract

Linezolid is an oxazolidinone antibiotic and is a last-line agent for treating infections caused by vancomycin-resistant *Enterococcus* (VRE). Limited work has been done to study the genomic epidemiology and population structure of linezolid-resistant *Enterococcus* (LRE) spp. in Victoria, Australia, and globally. We aimed to elucidate the genomic epidemiology and population structure of LRE in Victoria, Australia. We screened our local collection of *Enterococcus faecalis* and *Enterococcus faecium* for LRE from 2015 to 2023 and identified 15/349 (4.3 %) and 26/9,207 (0.28 %) *E. faecalis* and *E. faecium*, respectively, which had either or both phenotypic and genotypic resistance to linezolid. We next contextualized the Victorian LRE isolates with a global set of 684 *E. faecalis* and 324 *E. faecium* genomes from public databases. Integrated phylogenetic and epidemiologic data suggested that the LRE isolates in Victoria were likely introduced through multiple independent events from international travellers, with evidence of putative linear plasmids co-carrying *vanA* and linezolid resistance genes. We observed a predominance of the linezolid resistance-conferring *optrA* gene in *E. faecalis* irrespective of source origin in both the global and Victorian collections, whereas in *E. faecium*, *cfr* and *cfr*(B) occurred more frequently among clinical isolates, with no apparent geographical signatures. Our study provides a genomic snapshot of a large global collection of LRE isolates and establishes the epidemiological context for LRE circulating in Victoria, Australia.

## Data Summary

All short-read sequence data for Victorian isolates can be found under BioProject no. PRJNA856406. Raw FASTQ reads from Nanopore sequencing were deposited under nucleotide accessions SRR31690026 to SRR31690046. List of public isolates from the global dataset can be accessed through the Supplementary data files.

**Impact Statement**

Recent reports have described a rising prevalence of transferable resistance genes associated with reduced susceptibility to linezolid, a last resort drug for enterococcal infections. By studying the genome data of a large global collection of *Enterococcus faecalis* and *Enterococcus faecium*, we found that some linezolid resistance genes occurred more frequently in particular species, with certain genes showing a wider ecological range, for instance, the *optrA* gene in *E. faecalis*. The study also examined the genomic epidemiology of linezolid-*resistant E. faecalis* and *E. faecium* from patients in Victoria, Australia, spanning a wide study period since 2015, where linezolid-resistant *Enterococcus* (LRE) has not been widely reported in the Australian context. While we identified diverse linezolid resistance mechanisms in the local species, the overall prevalence of LRE in Victoria is currently low (<5 %). Integrating both genomic and patient data, we were able to identify putative linear plasmids co-carrying vancomycin and linezolid resistance genes and reconstruct an epidemiological link of linezolid resistance plasmids with international travellers. Our study provides a genetic background on the dissemination patterns and population structure of two prevalent and clinically relevant LRE species in a global and local context and demonstrates how genomics integrated with patient data can be used to infer potential sources of drug-resistant pathogens in a local clinical setting.

## Introduction

*Enterococcus faecium* and *Enterococcus faecalis* are commensals of the human intestinal tract but can cause serious, often healthcare-associated infections, being the third most common cause of healthcare-associated bacteraemia in the USA [1,2]. While most clinical *E. faecalis* retains susceptibility to amoxicillin, *E. faecium* is intrinsically resistant to many of these drugs and often reports high rates of multidrug resistance [3]. Resistance to first-line drugs such as beta-lactams and aminoglycosides has been increasingly documented over the last two decades, rendering them ineffective for therapy with serious clinical consequences.

Vancomycin has been the ‘gold-standard’ for the treatment of beta-lactam-resistant enterococci, but vancomycin resistance rates among clinical enterococci have risen in many countries, with few last-resort antimicrobials, including linezolid, left to treat vancomycin-resistant enterococcal (VRE) infections [4,5], leading to its classification as a high-risk pathogen by the World Health Organization (WHO) [6]. Linezolid is listed as a critically important antimicrobial by the WHO for VRE and acts by targeting the large (or 50S) ribosomal subunit, thereby inhibiting protein synthesis in Gram-positive bacteria. Resistance to linezolid in enterococci is conferred by chromosomal mutations or transferable genes. Mutations in the 23S rRNA gene are the most common mechanism conferring linezolid resistance in enterococci and are noted to develop during therapy with linezolid. Common mutations in the 23S rRNA include the nucleotide substitutions G to T at position 2576 (G2576T) and G to A at position 2505 (G2505A) based on *E. coli* numbering [7,8]. These mutations alter the conformation of the linezolid binding site on the large ribosomal subunit, leading to reduced linezolid susceptibility [9]. In *Enterococcus* spp., the 23S rRNA gene is present in multiple copies in *E. faecalis* (four copies) and *E. faecium* (six copies). A positive association has been demonstrated between the copy number of the mutated 23S rRNA allele and the level of linezolid resistance expressed [10]. Although most clinical LRE strains arise from 23S rRNA mutations, transferable genes conferring resistance to linezolid have been increasingly reported and characterized globally. These include genes encoding Cfr family proteins (*cfr*, *cfr*(B) and *cfr*(D)) which bind to and catalyse the methylation of the adenine nucleotide at position 2503 (A2503) of the 23S rRNA and genes encoding the ribosomal protection proteins OptrA and PoxtA [11,14].

Serious infections with VRE can only be treated with very few antimicrobials, namely linezolid, daptomycin and tigecycline. Pristinamycin is potentially active; however, access is a problem. Resistance to any of these last-line antimicrobials is concerning, as limited treatment options may render these infections untreatable [3]. Surveillance for linezolid-resistant enterococci (LRE) is currently not comprehensive or systematic in most countries and, as such, is usually reported as a subset of VRE from routine screening in clinical and public health laboratories. Outbreaks caused by LRE or linezolid-resistant VRE (LR-VRE) have not been widely reported, with the majority of cases involving *E. faecium* or *E. faecalis* documented in reports from only a few countries [15,16].

Australia has a high prevalence of VRE by global standards, with over 40% of bloodstream *E. faecium* reported to carry the vancomycin resistance genes *vanA* or *vanB* [3]. Most clinical VRE strains carry the *vanB* genotype, whereas *vanA* is the dominant genotype globally. In contrast, genes known to confer LRE in enterococci have not been well studied in terms of epidemiology, prevalence and distribution [3,17, 18]. A significant knowledge gap lies in understanding the epidemiological patterns and global dissemination of LRE strains carrying transferable linezolid resistance genes. This study was designed to provide an epidemiological context for the known linezolid resistance mutations (G2576T and G2505A) and genes *cfr*, *cfr*(B), *cfr*(D), *optrA* and *poxtA* in global and local Victorian *E. faecalis* and *E. faecium*.

## Methods

### Global *Enterococcus* dataset

We downloaded all Illumina paired-end reads (accessed on 14 April 2023) from the Sequence Read Archive (SRA) database for *E. faecalis* (*N*=12,292) and *E. faecium* (*N*=32,352). Selection criteria for SRA isolates are detailed in Fig. S1, available in the online Supplementary Material. From there, we identified and included 1,457 *E. faecalis* and 7,706 *E. faecium* that met the selection criteria with the following metadata: country, continent, isolation year, sample type or host. Additionally, we downloaded 613 *E. faecalis* and 212 *E. faecium* assemblies (draft or complete) with at least one known linezolid resistance gene (*cfr*, *cfr*(B), *cfr*(D), *optrA* and *poxtA*) from the National Center for Biotechnology Information (NCBI) Pathogen Detection database (accessed on 21 March 2023) and removed any SRA duplicates before incorporating these into the SRA dataset. In total, including sequencing reads from SRA and assemblies from NCBI Pathogen Detection, the global dataset included 2,070 *E. faecalis* and 7,918 *E. faecium*.

### Victorian *Enterococcus* dataset

The Critical Antimicrobial Resistance Alert (CARAlert) system was established as part of the Antimicrobial Use and Resistance in Australia (AURA) Surveillance System in Australia for real-time reporting of clinical pathogens resistant to critical last-line antimicrobials isolated nationwide [19]. The Microbiological Diagnostic Unit Public Health Laboratory (MDU-PHL) serves as a referral centre for CARAlert pathogens in the state of Victoria (population of over 6 million (25.7% of Australia’s total population) recorded on 31 March 2024 [20] despite being the smallest mainland state) including LRE and other antimicrobial resistance (AMR) pathogens collected across the state.

We selected isolates from a collection of 349 *E. faecalis* and 9,207 *E. faecium* referred to MDU-PHL between 2015 and 2023. Isolates were referred from public and private laboratories across Victoria, for diagnostic purposes, antimicrobial resistance testing, AMR snapshots and studies and mandatory referral of all *vanA* VRE isolates from December 2021 when it became a notifiable pathogen in Victoria. For our study, we selected a subset of these isolates using two search strategies: *E. faecium* and *E. faecalis* with a linezolid susceptibility phenotype (susceptible, intermediate or resistant) or a resistant genotype (representing isolates with one or more of these genes *cfr*, *cfr*(B), *cfr*(D), *optrA* or *poxtA*). Overall, we included 426 isolates from the search, including 379 *E. faecium* and 47 *E. faecalis*.

For antibiotic sensitivity testing, broth microdilution was performed using the Sensititre® broth microdilution assay (Thermo Fisher Scientific, Waltham, MA) with the GPN3F plate on 13 antimicrobials (ampicillin, ciprofloxacin, daptomycin, erythromycin, gatifloxacin, levofloxacin, linezolid, oxacillin, penicillin, quinupristin-dalfopristin, rifampicin, tetracycline and vancomycin) and interpreted based on Clinical and Laboratory Standards Institute (CLSI) M100 ed. 33 guidelines [21]. Strains with phenotypic resistance to three or more antimicrobial classes were classified as multidrug resistant.

### Short-read sequencing and trimming

All MDU-PHL enterococcal isolates were sequenced on the Illumina NextSeq platform with 2×150 bp paired-end reads. Genomic DNA extraction was carried out using the JANUS automated workstation (PerkinElmer, Waltham, MA, USA) and Chemagic magnetic bead technology (PerkinElmer, Waltham, MA, USA). DNA libraries were prepared using the Nextera XT kit according to the manufacturer’s instructions (Illumina Inc.). Adapter removal, trimming and quality control were performed on all MDU-PHL reads using fastp v0.23.2 [22] with default parameters and a threshold length of 36. Similarly, all SRA paired-end reads were trimmed and QC-checked using the same protocols as above.

### Long-read sequencing for Victorian isolates

Victorian enterococci with one or more linezolid resistance genes underwent long-read sequencing on Oxford Nanopore GridION (FLO-MIN106D R10). DNA extraction was performed using the Qiagen DNeasy Blood and Tissue kit according to the manufacturer’s protocols. DNA libraries were constructed with ligation sequencing using the Native Barcoding Kit 96 V14 (SQK-NBD114.96) according to the manufacturer’s instructions. Basecalling was performed using the built-in basecaller, Guppy v3.2.4, in GridION. Demultiplexing and adaptor trimming were performed using Porechop v0.2.4 (https://github.com/rrwick/Porechop) on the raw reads with default parameters. Trimmed reads were then passed through the long-read filter in Filtlong v0.2.1 (https://github.com/rrwick/Filtlong) with set parameters (minimum length of 1,000 bp and keep percent 95) to obtain a final read subset. These reads were then assembled *de novo* using Canu v2.2, Unicycler v0.5.0 (hybrid assembly with short reads) or Dragonflye v1.1.0 (https://github.com/rpetit3/dragonflye), and the best draft genome was chosen [23,24]. The qualities of the draft assemblies were assessed using QUAST v5.2.0 [25] to check for general contig properties, including contig length, GC content, N50 and total contig number.

### Bioinformatics analysis

All paired-end reads from SRA (global) and Victorian enterococci were assembled *de novo* with SPAdes v3.15.5 [26] and annotated using prokka v1.14.5 [27]. Species identification was carried out using Kraken2 v2.1.3 [28] using trimmed FASTQ reads as input with default parameters. *In silico* multilocus sequence typing (MLST) was performed on all isolates using mlst v2.23.0 (https://github.com/tseemann/mlst) against PubMLST databases for *E. faecalis* and *E. faecium*. Putative antimicrobial resistance genes with >90 % sequence coverage were identified by running abritAMR v1.0.11 against the AMRFinderPlus database [29]. LRE-Finder v1.0 [30] was run with default parameters using raw FASTQ files to estimate the presence and copy number of G2576T and G2505A substitutions on the 23S rRNA alleles. Thresholds used to define the copy number of mutated alleles are available in Table S6.

### Genetic characterization of linezolid resistance genes

To understand the genetic context of linezolid resistance genes in Victorian enterococci, we characterized the flanking regions of these genes from hybrid assemblies generated from Illumina and Nanopore reads. The genomic location of these genes was identified by aligning the hybrid assemblies against reference nucleotides for *cfr*, *cfr*(B), *cfr*(D), *optrA* and *poxtA* (see Table S9) using the nucleotide blast (blastn) option in Bandage v0.9.0 [31]. Flanking regions of the genes were extracted in Geneious Prime 2023.0.4 and aligned in Easyfig v2.2.2 [32]. For putative plasmid assemblies with linezolid resistance genes, contigs were screened for plasmid-associated genes (plasmid replication gene, relaxase gene and origin of replication), transposable elements and AMR-related genes using Mob-typer as part of the MOB-suite v3.0.3 utility [33] and bakta v1.8.1 with default parameters [34].

### Phylogenetic analysis and tree construction

Maximum likelihood trees based on core genome SNPs were constructed for both global and Victorian isolates. All core genome alignment and variant calling were performed using snippy and snippy-core v4.4.5 (https://github.com/tseemann/snippy) with species-specific reference genomes (*E. faecalis* accession no. NC_004668 and *E. faecium* accession no. NZ_CP027501). The resulting alignment was used as input for IQ-TREE v2.0.6 [35] to construct a maximum likelihood tree using the generalized time-reversible model of evolution (GTR+G4) with 1,000 bootstraps. Additionally, genetic clustering using the Bayesian algorithm was performed for the Victorian dataset using the rhierbaps package v1.1.4 in R v4.2.2 [36].

### Data visualization

All long-read assemblies, including plasmid contigs, were visualized in Bandage to check for contig circularization and copy number of the 16S rRNA gene (four in *E. faecalis* and six in *E. faecium*). Regions of synteny between similar linezolid resistance genes in different isolates were compared and visualized using the gggenomes package v1.0.0 in R v4.2.2. Whole plasmid map was visualized using the plasmapR package v0.3.1 in R or by blast Ring Image Generator [37] for plasmid sequence comparison. All phylogenetic trees were visualized in R using the ggtree v3.6.2 package.

## Results

### Linezolid resistance mechanisms vary in Victorian enterococci

**Fig. 1.**
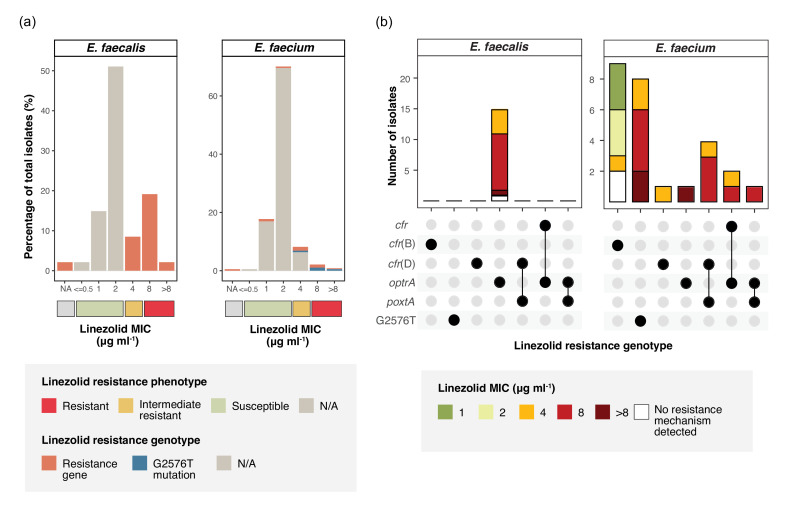
Phenotypic and genotypic linezolid susceptibility profiles of *E. faecalis* and *E. faecium* from Victoria, Australia (2015–2023). (**a**) Linezolid MIC distribution correlated with resistance genotype (gene or mutation) for all *E. faecalis* (*N*=47) and *E. faecium* (*N*=379). (**b**) Upset plot showing the MIC variations for gLR*E. faecalis* (*N*=15) and gLR *E. faecium* (*N*=26) carrying one or more linezolid resistance determinants including mutation and genes. MIC results interpreted based on CLSI M100 ed. 33, 2023 [21].

We compiled *E. faecalis* (*N*=349) and *E. faecium (N*=9,207) sequences from Victoria, Australia, between 2015 and 2023 to assess LRE rates and genetic mechanisms in response to detecting LRE in our jurisdiction (Fig. S2). Among the dataset, we noted a higher proportion of LRE based on phenotype or genotype in *E. faecalis* (15/349, 4.3%) compared to *E. faecium* (26/9207, 0.28%). We screened the sequences for the presence of linezolid resistance genes and 23S ribosomal mutations. In the 15 LRE *E. faecalis*, we only identified *optrA*, but in *E. faecium* (*N*=26), we identified numerous genetic mechanisms, including *cfr* (*N*=2)*, cfr*(B) (*N*=9), *cfr*(D) (*N*=5), *optrA* (*N*=4), *poxtA* (*N*=5) and the G2576T mutation (*N*=8) with seven isolates co-carrying more than one resistance gene (Fig. 1b). The majority of *E. faecium* also carried the *vanA* operon (19/26, 73.1%), with only five *vanB* isolates and no *vanM* detected, despite the former being more dominant in Australian VRE [3].

All phenotypic linezolid resistance (MIC ≥8 µg ml^−1^) identified from Victorian enterococci was explained by known genetic determinants (Fig. 1a). The *optrA* gene in *E. faecalis* conferred a minimum inhibitory concentration (MIC) of ≥4 µg ml^−1^ corresponding to an intermediate or resistant phenotype. In *E. faecium*, *cfr*(B) was found frequently but did not confer phenotypic resistance (Fig. 1b). In one of these *E. faecium* isolates, the *cfr(B*) gene was located in close proximity to the Tn*916-tet(M*) transposon (Fig. S3). The phenotype of the G2576T mutation was determined by copy number, with at least two mutated copies of the 23S gene required for MIC of ≥4 µg ml^−1^ (Fig. S4). The other identified mechanisms in *E. faecium* all conferred resistance (Fig. 1b). Given that not all isolates with genotypic resistance were phenotypically resistant to linezolid, we herein refer to all enterococci with genotypic resistance (carrying resistance gene or mutation) as genomic LRE (gLR *E. faecalis* and gLR *E. faecium*).

### Linezolid resistance genes can be encoded on putative linear plasmids

Our initial bioinformatics analysis on the short-read sequences predicted that several of the LRE genes were encoded on plasmids. To confirm this, we undertook long-read sequencing on a subset of isolates (10 gLR *E. faecalis* and 11 gLR *E. faecium*) that were shown to carry one or more linezolid resistance genes, including 10 gLR *E. faecalis* and 11 gLR *E. faecium*.

Using long-read sequence data, we confirmed the chromosomal localization of *optrA* genes in all 10 *E. faecalis* isolates from long-read sequencing (Fig. S5). Using these as references, we predicted that three more Illumina-sequenced *E. faecalis* also carried *optrA* on the chromosome. Out of the 11 gLR *E. faecium* sequenced, five carried linezolid resistance genes on the chromosome, while the remaining six had plasmid-borne resistance genes. Two of the six *E. faecium* isolates carried linezolid resistance genes on circular plasmids (Fig. S6).

**Fig. 2.**
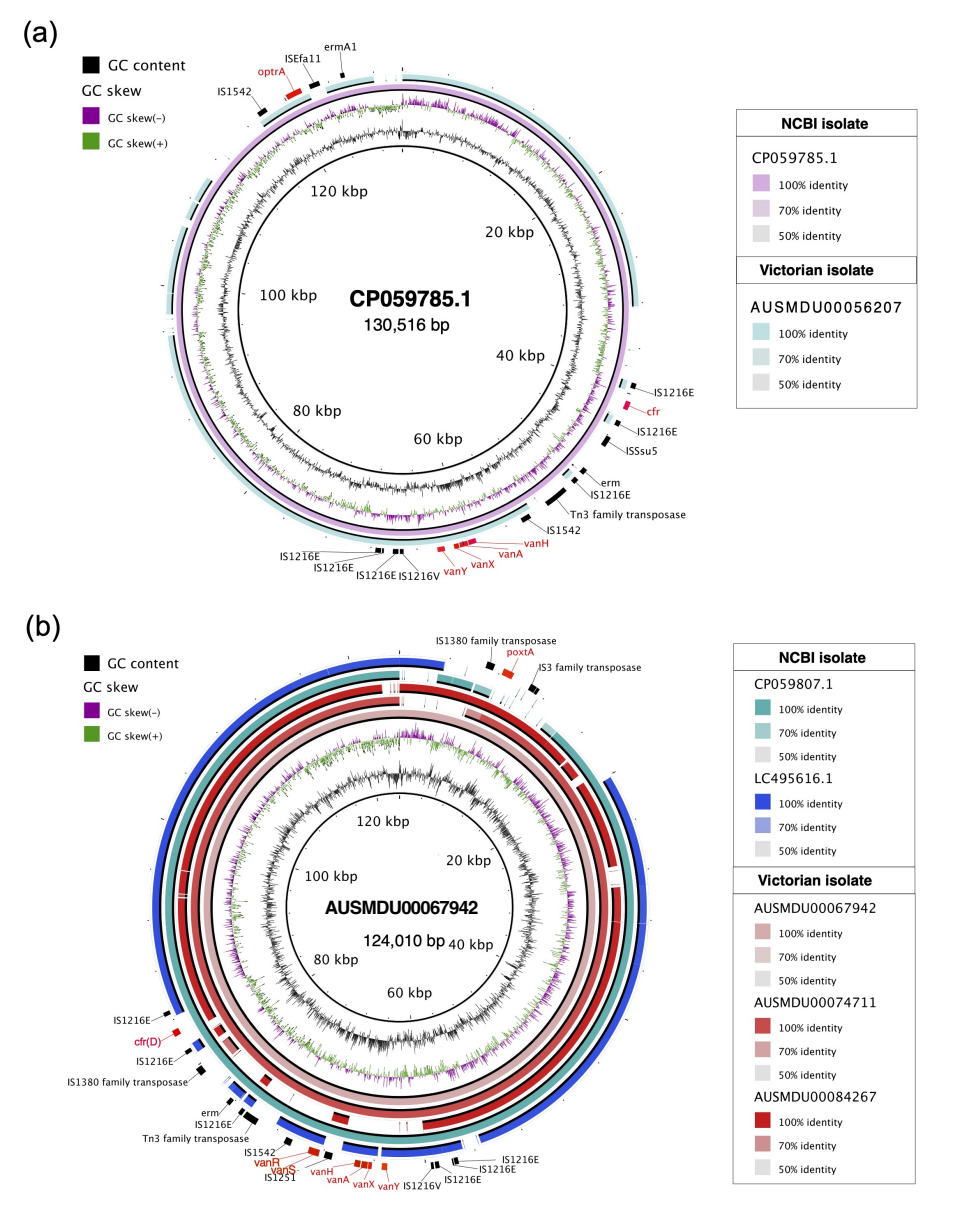
Comparative plasmid maps of Victorian *E. faecium* pELF1-like plasmids carrying vancomycin (*vanA*) and linezolid resistance genes. Each solid band represents a single plasmid. Resistance genes were annotated in red. (**a**) Multiple alignment of plasmid co-carrying *vanA* and *cfr/optrA* from Australian *E. faecium* (light blue) and pELF1-like plasmids from India (GenBank accession: CP059785.1) with the latter used as the reference. From innermost to outermost: GC content, GC skew, CP059785.1 and AUSMDU00067942. (**b**) Multiple alignment of plasmids co-carrying *vanA* and *cfr(D)/poxtA*. Plasmids from Australia *E. faecium* (red) were aligned against pELF1-like plasmids from India and Japan (GenBank accessions: CP059807.1 and LC495616.1, respectively). AUSMDU00067942 was used as the reference. From innermost to outermost: GC content, GC skew, AUSMDU00067942, AUSMDU00074711, AUSMDU00084267, CP059807.1 and LC495616.1.

Four gLR *E. faecium* were found to harbour putative linear plasmids which co-carried multiple linezolid resistance genes (Fig. 2). Linearity was confirmed via alignment of Nanopore reads to the plasmid contig (Fig. S7) [38]. Using blastn, we found that these plasmids closely matched (≥50% coverage and identity) the pELF1 and pELF1-like linear plasmids from India, Japan and Ireland [39,41]. All four plasmids share a highly similar backbone with the same plasmid replication initiation RepB gene but differ in their AMR gene content, with three of the plasmids also co-carrying the *vanA* gene cluster. Epidemiological data revealed travel histories of these patients from India, suggesting repeated importations of the plasmids into Victoria, rather than clonal expansion.

### Victorian LREs are genetically diverse and are not phylogenetically clustered

**Fig. 3.**
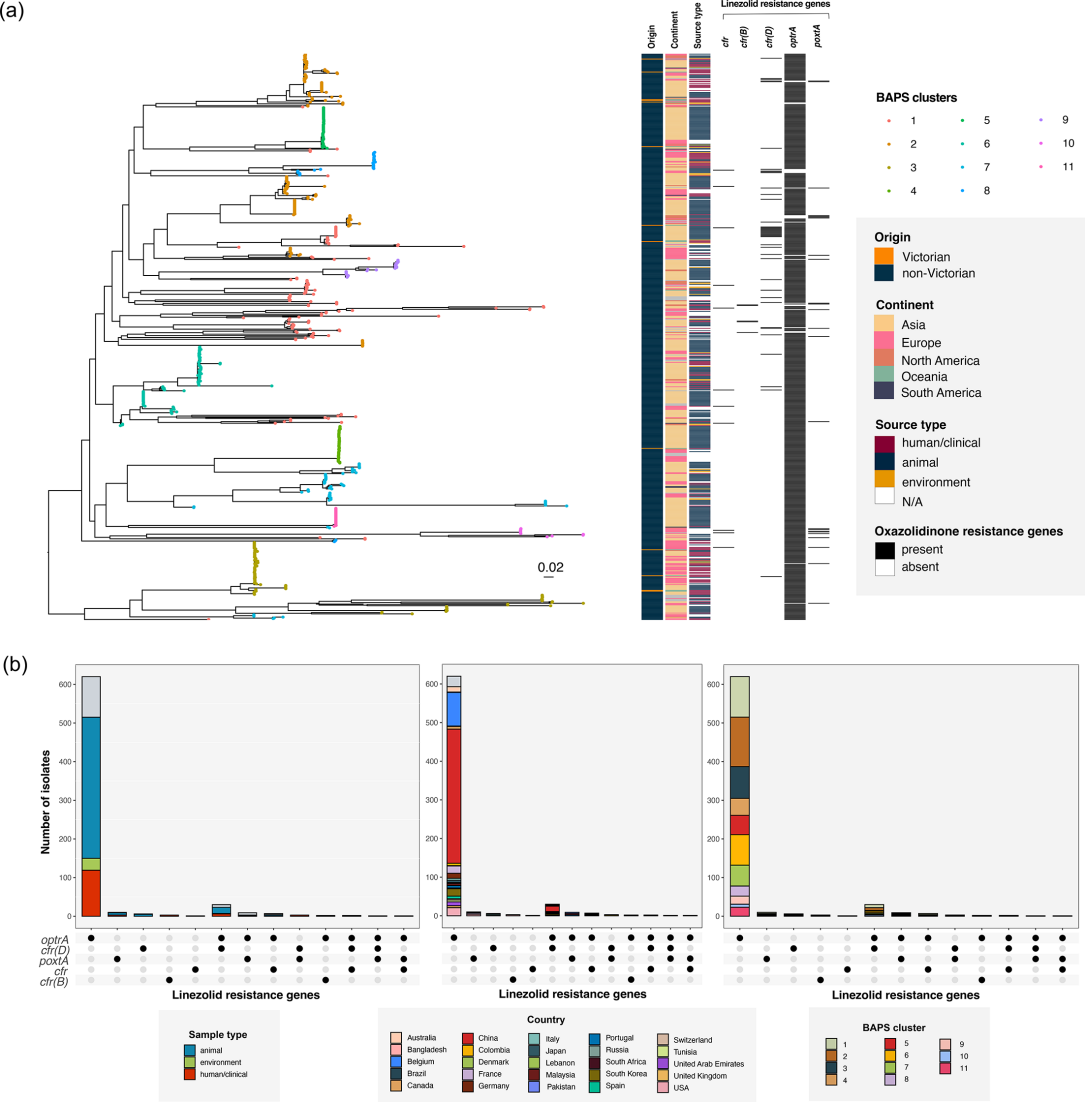
Phylogenetic tree and metadata for global (*N*=684) and Victorian (*N*=15) gLR *E. faecalis*. (**a**) Midpoint-rooted maximum likelihood tree for global gLR *E. faecalis* based on core genome SNP alignment. Tips were coloured according to the BAPS cluster. The heatmap panel shows the origin, continent, source type, and linezolid resistance genes (from left to right: *cfr, cfr*(B)*, cfr*(D)*, optrA* and *poxtA*). Victorian isolates (orange) were annotated in the first heatmap column. (**b**) Breakdown of isolate metadata (sample type, country of origin and BAPS cluster) for each linezolid resistance gene type.

Analysis of the sequence types (STs) of the LRE isolates suggests no single circulating clone in Victorian hospitals (Figs S8 and S9). To further confirm our hypothesis of non-clonal dissemination of LRE in Victoria and to understand their phylogenetic context, we compared the Victorian LRE to the global gLRE sequences (Figs3  4). The global dataset includes genomes from SRA and NCBI carrying one or more linezolid resistance genes *cfr*, *cfr*(B), *cfr*(D), *optrA* and *poxtA*. In total, we included 684 *E. faecalis* and 324 *E. faecium* in our global dataset, with a higher overall prevalence in *E. faecalis* (684/2,070 (33.0 %) gLR) than *E. faecium* (324/7,918 (4.1 %) gLR from screening the public databases (see section ‘Population structure of global gLRE’ below for results on population structure).

**Fig. 4.**
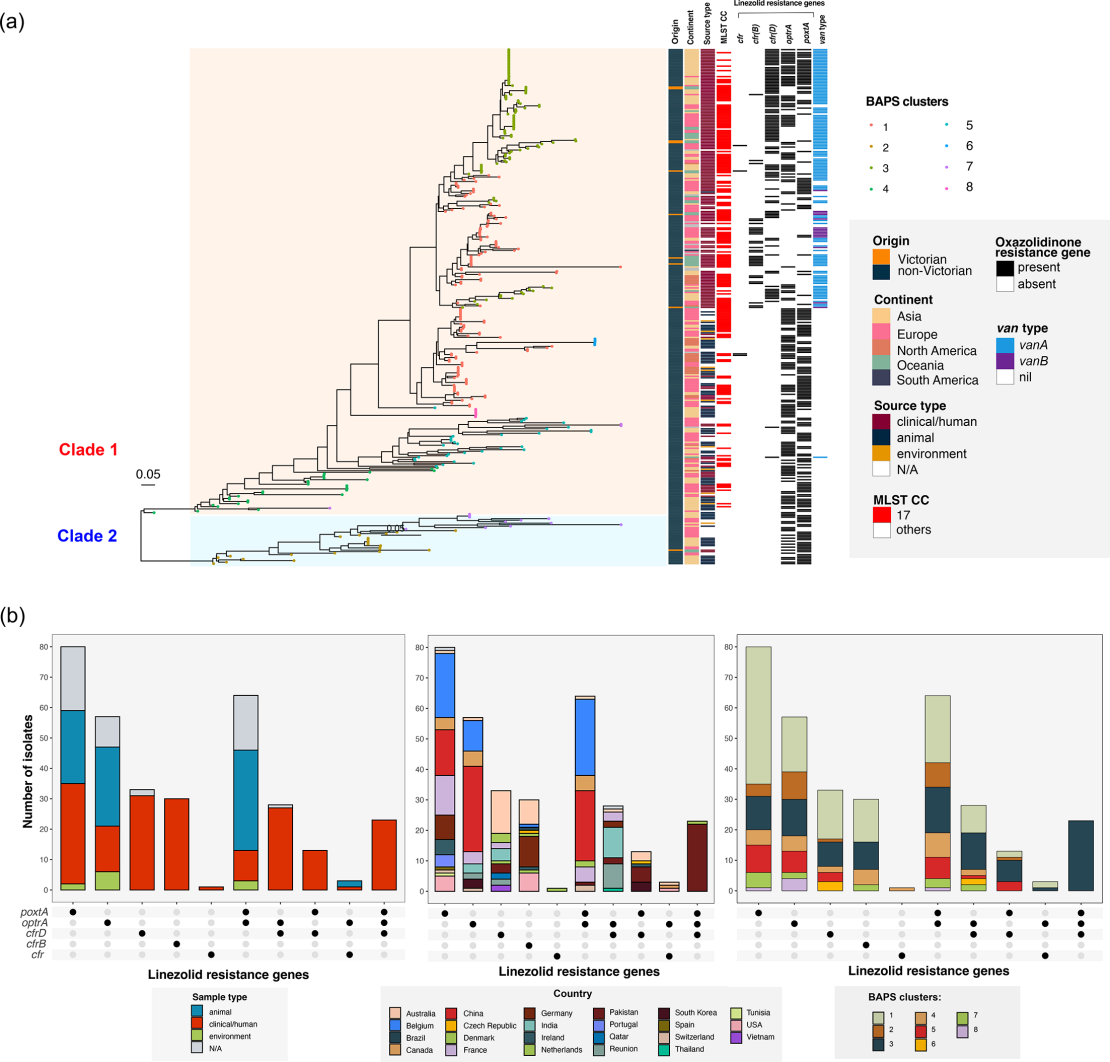
Phylogenetic tree and metadata for global (*N*=324) and Victorian (*N*=26) gLR *E. faecium*. (**a**) Midpoint-rooted maximum likelihood tree for global gLR *E. faecium* based on core genome SNP alignment. Tips were coloured according to the BAPS cluster. The heatmap panel shows the origin, continent, source type, ST, MLST clonal complex (CC), linezolid resistance genes and *van* type. Victorian isolates (orange) were annotated in the first heatmap column. (**b**) Breakdown of isolate metadata (sample type, country of origin and BAPS cluster) for each linezolid resistance gene type.

### Global LRE mechanisms varied across species

Despite an overall higher proportion of gLR *E. faecalis* compared to gLR *E. faecium* globally, the resistance mechanism was more uniform in gLR *E. faecalis* with *optrA* being the primary resistance determinant (659/684, 96.3%, Fig. 3b). In gLR*E. faecium*, a considerably higher proportion of non-*optrA* genes (*poxtA*: 176/324 (54.3 %), *optrA*: 172/324 (53.1 %), *cfr*(D): 97/324 (30.0 %)) were detected with *cfr*(D), *optrA* and *poxtA* frequently co-carried in the same isolate (Fig. 4a). The *cfr*(B) gene was almost exclusively found among clinical *vanA* gLR *E. faecium*.

We estimated that the G2576T mutation occurred in only a small proportion of the global gLR *E. faecalis* (0.69%) and gLR *E. faecium* (2.75%) from SRA (*E. faecalis*=1,457 and *E. faecium*=7,706) using LRE-finder. A small proportion of the G2576T *E. faecium* (7/212, 3.3%) co-carried linezolid resistance genes. The mutation occurred most frequently in two out of four (*E. faecalis*) or six (*E. faecium*) copies of the 23S rRNA allele (Fig. S10).

### LRE genes are present globally

We next assessed the source origin and geographical distribution of all linezolid resistance genes from our study dataset in the global gLRE dataset (Figs3b  4b). Overall, the global linezolid resistance gene distribution displayed a stronger overlap with sample type than geography. Within the global gLRE dataset, we saw a greater proportion of clinical/human isolates (54.3%) for gLR *E. faecium* vs. animal/environmental (63.5%) isolates for gLR*E. faecalis*. In gLR *E. faecalis*, the prevalence of *optrA* was high in both clinical/human (92.1%) and animal/environmental isolates (96.8%). Comparatively, the *cfr* and *cfr*-like genes (6.9–13.4 %) and *poxtA* (1.8–7.1 %) occurred at very low prevalence in gLR *E. faecalis* regardless of sample type. Among gLR *E. faecium*, the *cfr* gene occurred more commonly in clinical (68.2%) than in animal/environmental (2.1%) isolates. In contrast, the *optrA* and *poxtA* genes occurred more commonly among animal/environmental isolates (64.6–72.9 %, respectively) than in clinical/human isolates (41.5–42.6%).

**Fig. 5.**
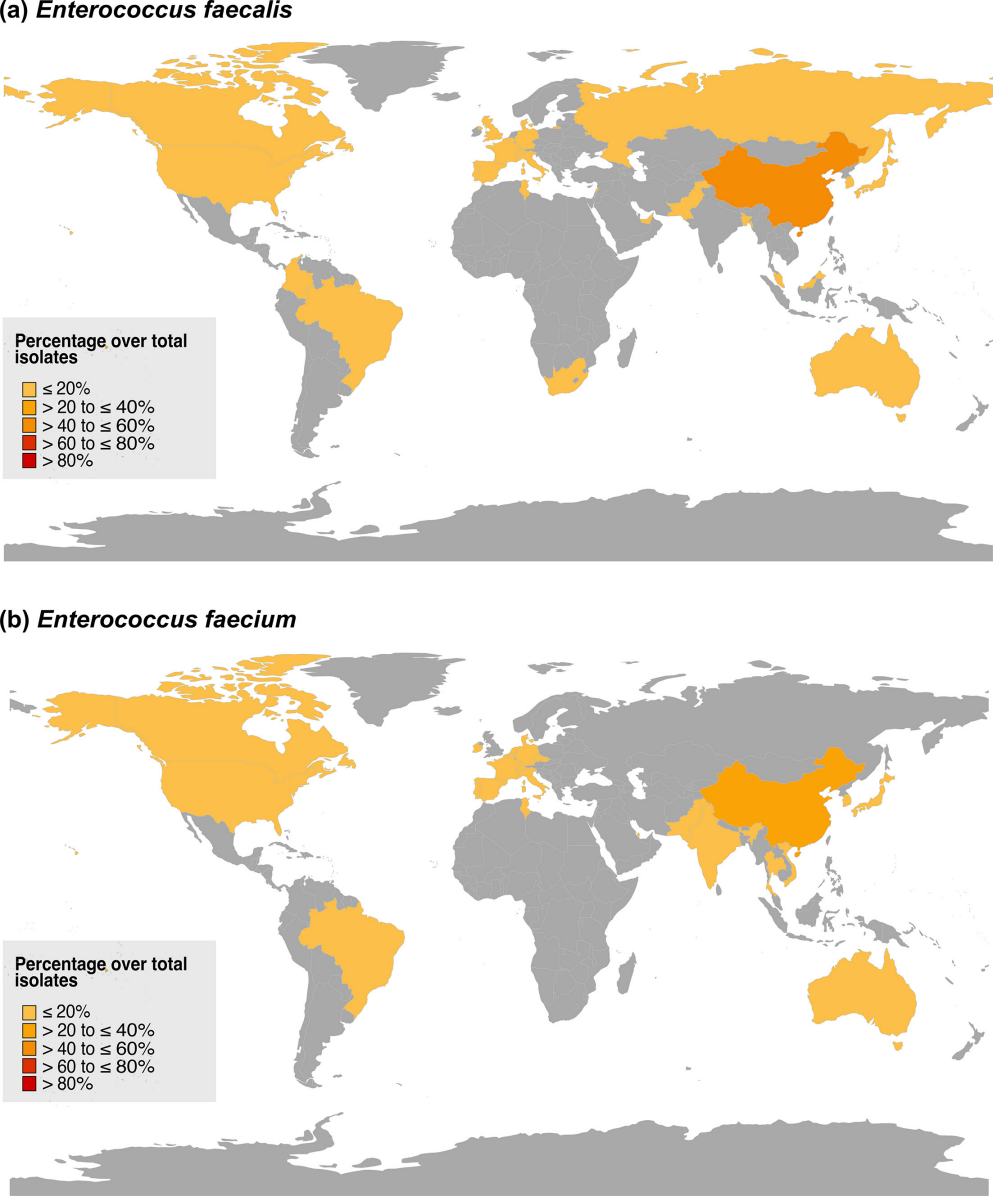
Geographical snapshot of known linezolid resistance genes *cfr*, *cfr(B)*, *cfr(D)*, *optrA* and *poxtA* identified from public databases for (**a**) gLR *E. faecalis* and (**b**) gLR *E. faecium*. The dataset includes both SRA reads and NCBI assemblies. Countries are coloured by percentage over total isolates for *E. faecalis* (*N*=684) and *E. faecium* (*N*=324).

Both gLR *E. faecalis* and gLR *E. faecium* sequences were found in Asia, Europe, North and South America, but there was limited data available for Africa (Fig. 5). Most gLR *E. faecalis* were identified from Asia (62.3%), whereas most gLR *E. faecium* were from Europe (44.8%). For gLR *E. faecalis*, a large proportion (25.8%) were non-clinical *optrA* isolates sampled from China under two BioProject accessions (PRJNA856057 and PRJNA896765) involving single-site LRE sampling, partly explaining the observed epidemiological bias.

### Population structure of global gLRE

Core SNP phylogenies with Bayesian Analysis of Population Structure (BAPS) cluster annotation were constructed for the global gLRE species. Overall, gLR *E. faecalis* isolates displayed a heterogeneous population structure divided into 11 BAPS clusters (Fig. 3a). The *optrA* isolates belonged to multiple BAPS clusters, indicating that the gene was disseminated in multiple lineages. For gLR *E. faecium*, we identified a total of eight BAPS clusters which divide into two major clades 1 and 2 (Fig. 4a). The larger clade 1(291/324 isolates) consists mostly of clinical/human CC17 isolates, whereas clade 2 (33/324 isolates) isolates were mostly animal or environmental. We further compared the gLR *E. faecium* clades from this study with the previously established *E. faecium* clades based on core gene SNP-based phylogeny – clade A1 (clinically associated lineage), clade A2 (clinical lineage intermixed with animal isolates) and clade B (community lineage) [42]. From there, we observed that the gLRE clade 2 isolates (33/324, 10.2%) from our study corresponded mostly to clade A2 strains, whereas the majority of the clade 1 isolates, including those with and without *vanA*, aligned with the clinically associated isolates from clade A1 [42] (Table S8).

## Discussion

Globally, there have been numerous studies that described LRE in clinical and livestock settings [41,43,46], but similar studies were limited in Australia [17,18] that spans a considerable study period and no reports have been published in Victoria, Australia. Here, we utilize both genomic and epidemiological data to investigate the population structure of *E. faecalis* and *E. faecium* with transferable linezolid resistance genes in Victoria, Australia, as well as study the population structure of a large global collection of LRE species using genomes gathered from public databases. Both epidemiological and phylogenetic data suggest multiple, independent introductions of LRE strains into Victoria rather than through local clonal expansion. The majority of the STs identified for gLR *E. faecium* were prevalent clinical strains circulating in Australia, such as ST78, ST80, ST796, ST1421 and ST1424 [3]. No considerable overlap was observed between phylogenetic (BAPS) clusters and linezolid resistance genes in both species globally, suggesting that these genes were not limited to a specific lineage but rather spread through multiple lineages in different geographical settings, likely through horizontal transfer via plasmids or transposons.

Overall, our findings align with previous studies demonstrating the *optrA* gene as the predominant resistance determinant in *E. faecalis* [47,49]. A stronger overlap was observed between linezolid resistance genes and source type than geography for both species, indicating that different genes could have been maintained under different selective pressures and subsequently disseminated within a given niche. The widespread dissemination of *optrA* in both global and Victorian LR*E. faecalis* was likely due to (i) the co-selection of *optrA* with the phenicol transporter gene *fexA*, given their frequent co-localization on the same mobile genetic element; (ii) the widespread use of phenicols as growth promoters in livestock [48,50, 51]; and (iii) the presence of *optrA* on sex pheromone-responsive plasmids, which are generally more widespread in *E. faecalis* [52]. In contrast, Cfr family genes, particularly *cfr* and *cfr*(B), occurred more frequently among clinical *E. faecium* isolates, suggesting that these genes could have been selected under a hospital environment. Given that other Cfr gene variants have also been identified in *Clostridiodes difficile*, it is possible that the *cfr* and *cfr*(B) genes could have been acquired from a non-enterococcal host species that shares the same niche (e.g. human gut) as enterococci [53], as observed previously with the *vanB* gene [54].

A considerable proportion of global LRE overlapped with *vanA* carriage, implying that vancomycin use could act as a selective force or underlying sampling bias in the global LRE dataset. Further, we also identified evidence of putative linear plasmids carrying *vanA* and linezolid resistance genes introduced into Australia through international travellers. Similar linear plasmids have been described in *E. faecium* from Japan, Ireland and India [39,41]. The coexistence of *vanA*-mediated resistance to glycopeptides and linezolid resistance raises concerns about potentially untreatable VRE infections, with daptomycin and tigecycline the only remaining treatment options for serious infections.

In Australia, *vanB* is the dominant vancomycin resistance genotype in clinical *E. faecium* strains, which are likely to be predominantly locally transmitted. Our findings suggest that most cases of linezolid-resistant enterococci in Victoria, Australia, were imported, where these isolates had the *vanA* gene, explaining linezolid resistance to be more common in *vanA*-containing strains. In one *E. faecium* isolate, we observed the integration of the *cfr*(B) gene in close proximity to the Tn*916-tet(M*) transposon. Previously, this transposon has been associated with the dissemination of the tetracycline resistance gene *tet(M*) with evidence supporting within-host transposition [55]. To the best of our knowledge, this was the first description of the genetic association between *cfr*(B) and *tet(M*) on a Tn*916*-like transposon in enterococci and signifies the potential of co-transmission of resistance genes to multiple antimicrobial classes on the same transposable element.

We identified several limitations in this study. First, both our local and global gLRE datasets displayed strong sampling biases. The local Australian LRE isolates represented only clinical human samples collected after 2015 in a single state, with *E. faecium* isolates strongly skewed towards VRE. On the other hand, the majority of the global gLRE sequences were from China, reflecting regional biases. Within each global gLRE species, most *E. faecalis* were isolated from animal sampling, in contrast to *E. faecium* from clinical human samples. The second limitation was the lack of phenotypic resistance data for the global gLRE isolates, which, if present, allows us to assess the phenotypic distribution and diversity of each resistance genotype. Future studies could expand to look at Australian LRE from a wider ecological context from One Health sampling to provide a more comprehensive representation in the region.

Our study highlights how genomics and epidemiology data are important in understanding LRE epidemiology in a global and local context. Using long read sequencing, we were able to elucidate the genetic background of linezolid resistance genes in two clinically significant enterococcal species, *E. faecalis* and *E. faecium* in Victoria, Australia. Our findings suggest a persistent One Health reservoir for transferable linezolid resistance genes and their capacity to disseminate across ecological barriers, highlighting the importance of genome-based surveillance in public health interventions and transmission of drug-resistant pathogens.

## Supplementary material

10.1099/mgen.0.001432 Uncited Supplementary Material 1.

10.1099/mgen.0.001432 Uncited Supplementary Material 2.

## Abbreviations

AMR: antimicrobial resistance
BAPS: Bayesian Analysis of Population Structure
CARAlert: Critical Antimicrobial Resistance Alert
CLSI: Clinical and Laboratory Standards Institute
gLRE: genomic LRE
LRE: linezolid-resistant *Enterococcus*
MDU-PHL: Microbiological Diagnostic Unit Public Health Laboratory
MIC: minimum inhibitory concentration
MLST: multilocus sequence type
NCBI: National Center for Biotechnology Information
SRA: Sequence Read Archive
ST: sequence type
VRE: vancomycin-resistant *Enterococcus*
WHO: World Health Organization
