# Supplementary material for: Genomics to understand the global landscape of linezolid resistance in Enterococcus faecium and Enterococcus faecalis
Source: Microb Genom. 2025 Jun 18;11(6):001432. doi: 10.1099/mgen.0.001432 (PMC12177158; doi:10.1099/mgen.0.001432)
Supplement: Uncited Supplementary Material 1. [file mgen-11-01432-s001.pdf]

Genomics to understand the global landscape of linezolid resistance in *Enterococcus faecium* and *Enterococcus faecalis*

Jia Qi Beh[2], Diane S. Daniel[1], Louise M. Judd[2,3], Ryan R. Wick[2], Peter Kelley[4,6], Katie M. Cronin[5], Norelle L. Sherry[1,2,7], Benjamin P. Howden[1,2,3,7], Christopher H. Connor[2#] and Jessica R. Webb[2,3#]

1. Microbiological Diagnostic Unit Public Health Laboratory, Department of Microbiology and Immunology at the Peter Doherty Institute for Infection & Immunity, The University of Melbourne, Melbourne, Victoria, Australia.
2. Department of Microbiology and Immunology at the Peter Doherty Institute for Infection and Immunity, The University of Melbourne, Melbourne, Australia.
3. Centre for Pathogen Genomics, University of Melbourne, Melbourne, Victoria, Australia.
4. Department of Infectious Diseases, Peninsula Health, Frankston, Victoria, Australia.
5. Department of Microbiology, Royal Melbourne Hospital, Melbourne, Australia.
6. Department of Microbiology, Eastern Health, Box Hill, Victoria, Australia.
7. Department of Infectious Diseases & Immunology, Austin Health, Heidelberg, Victoria, Australia.

#co-senior

Corresponding author: Benjamin Howden; bhowden@unimelb.edu.au

Supplementary Figures

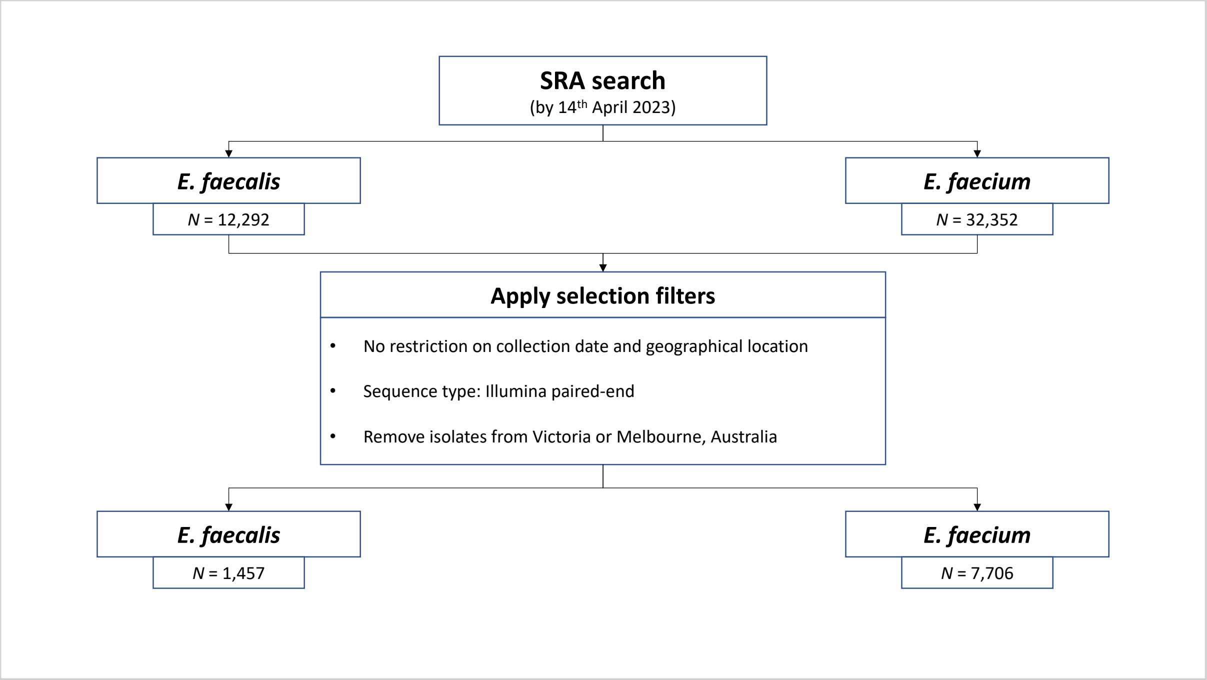

Figure S1. Selection workflow for SRA global enterococci.

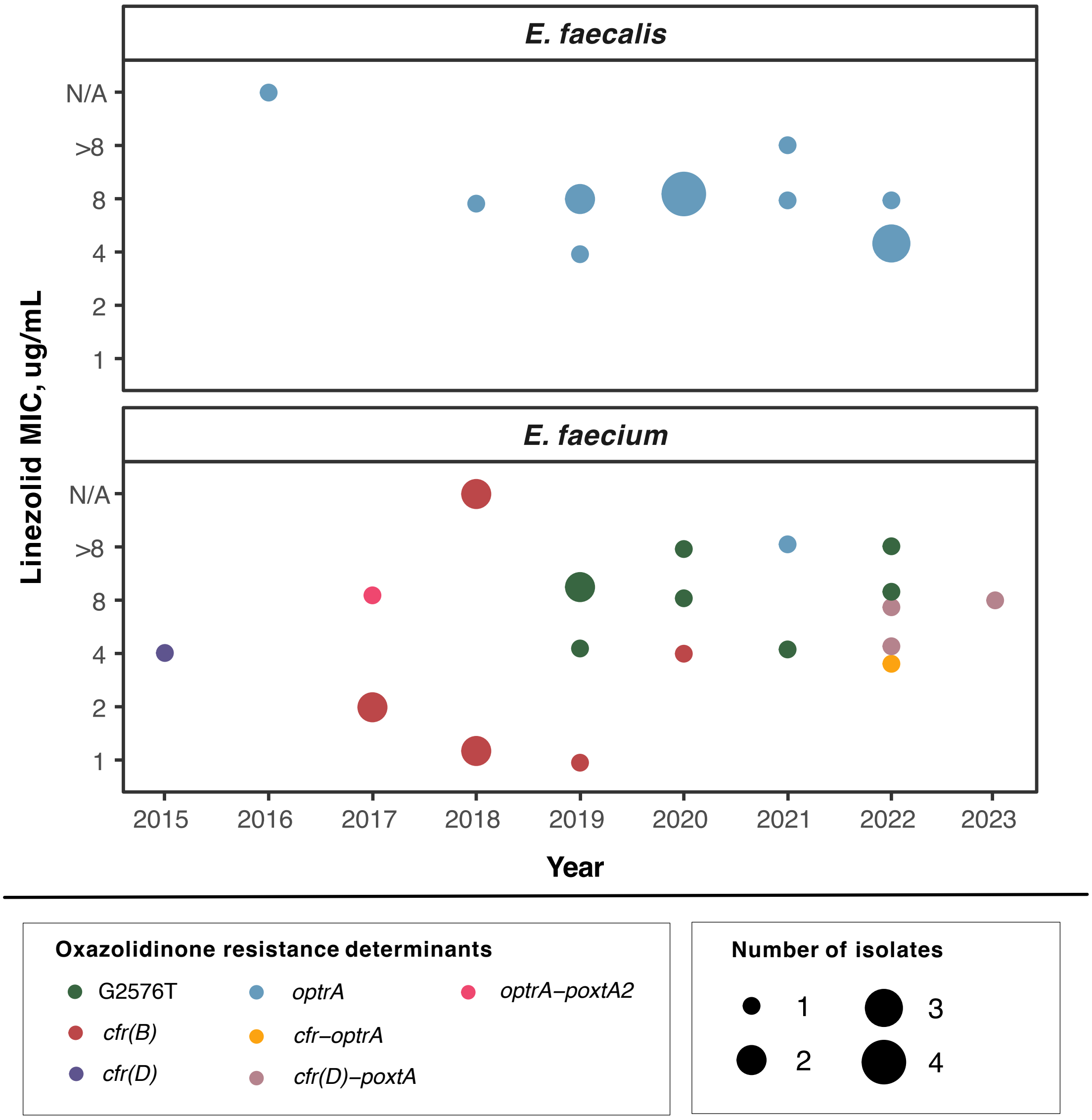

Figure S2. Linezolid genotype and phenotype distribution in Victorian LRE during the study period (2015-2023). Data points were coloured according to oxazolidinone resistance genes. The size of dot is proportional to the number of isolates corresponding to each isolation year and source hospital.

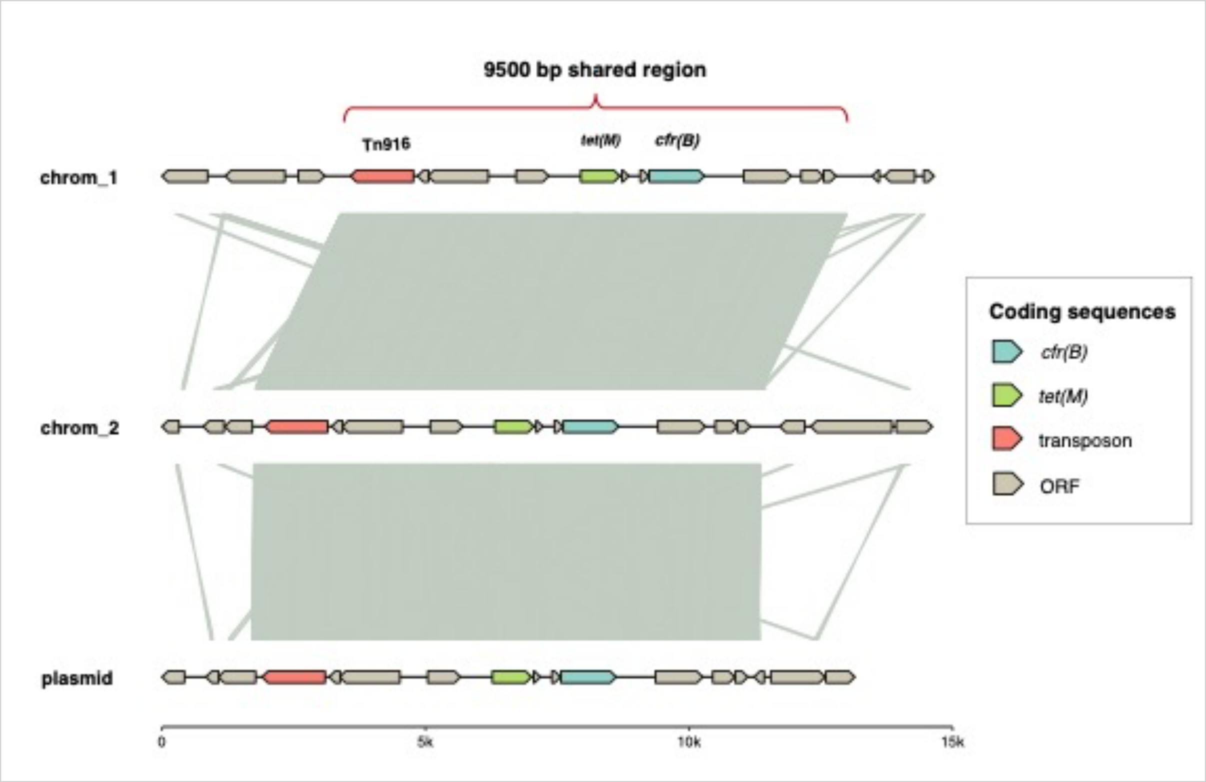

Figure S3. Linear representation of the *cfr(B)* flanking regions in *E. faecium* isolate AUSMDU00046081 (ST-2405).

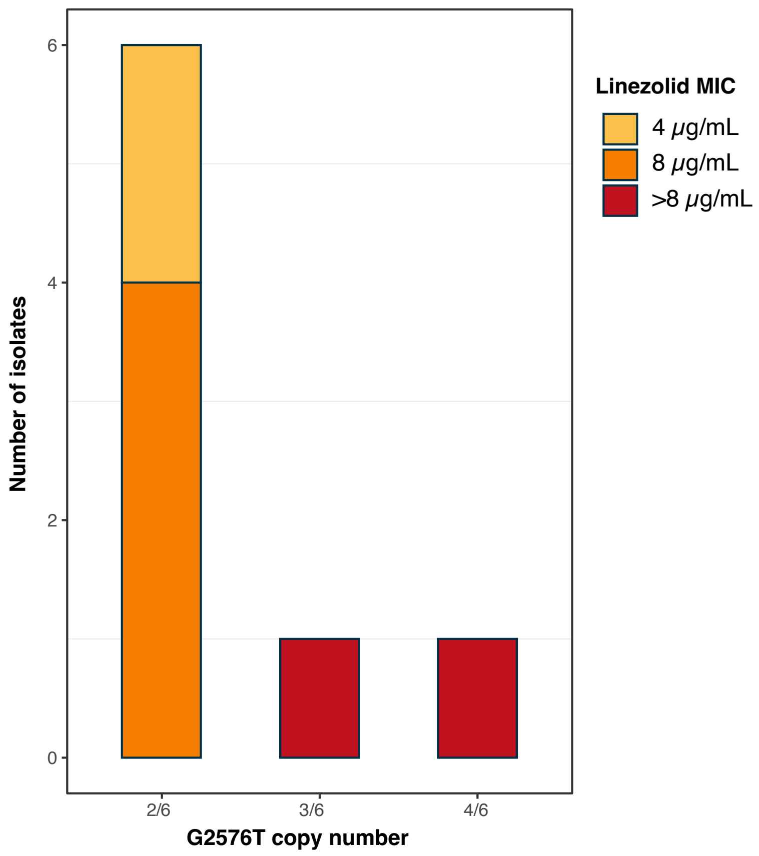

Figure S4. Correlation of G2576T mutation with linezolid MIC in the Victorian *E. faecium* isolates. We identified only isolates with two (2/6), three (3/6) and four (4/6) out of six mutated 23S rRNA alleles.

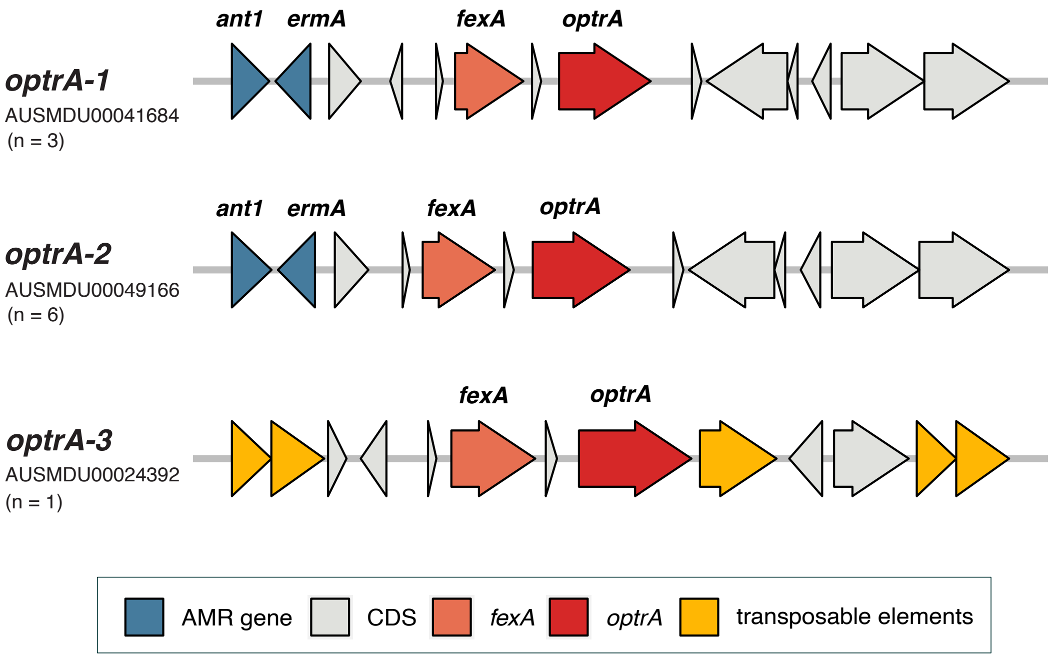

Figure S5: Genetic environment of *optrA* genes in Nanopore sequenced *E. faecalis* (*N* = 10) in Victoria. ORFs were annotated according to gene type.

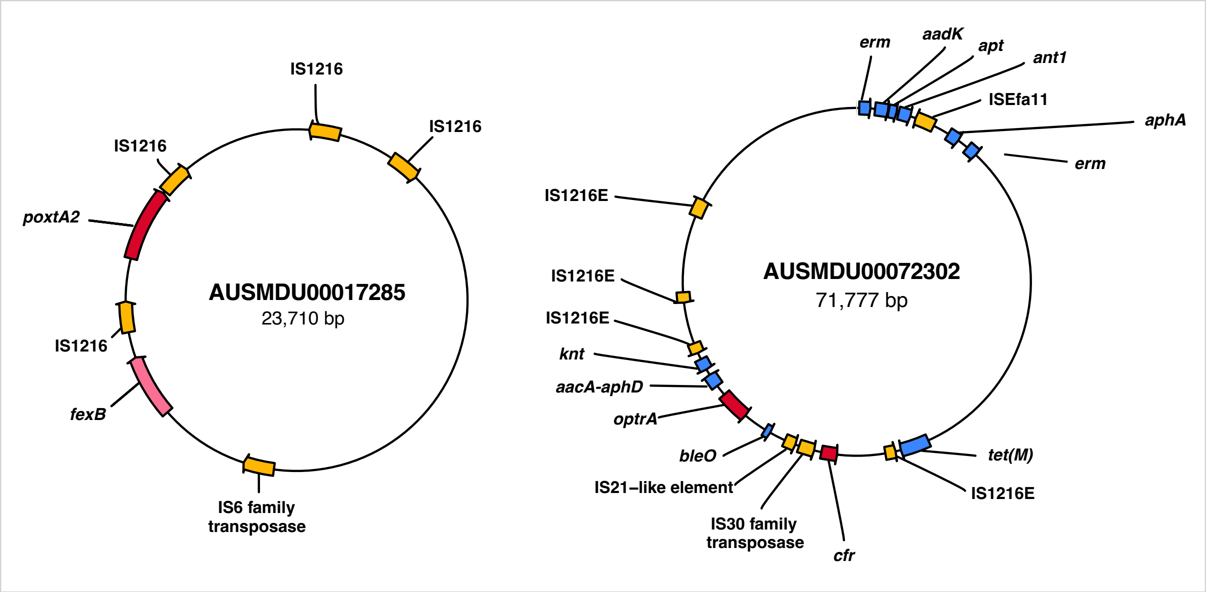

Figure S6. Circular plasmids carrying linezolid resistance genes in Victoria Australian *E. faecium*.

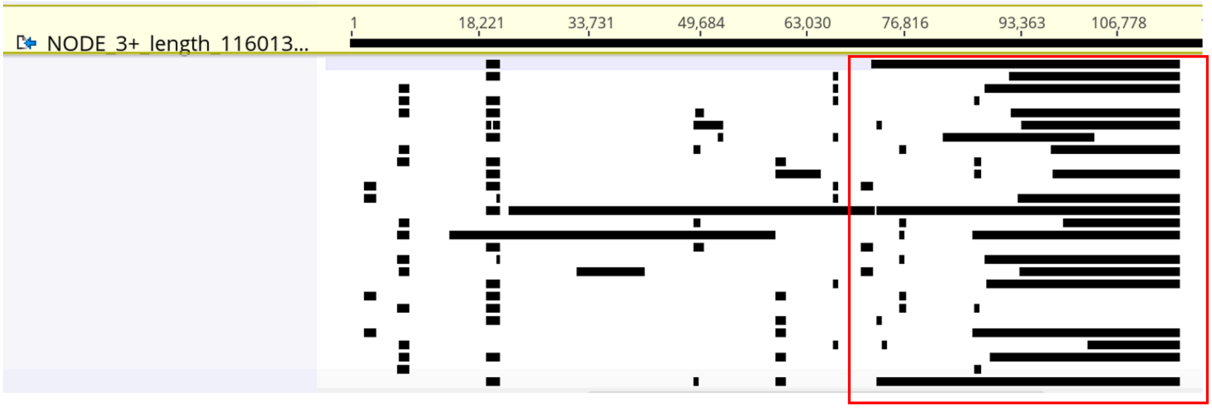

Figure S7. Alignment of Nanopore reads of isolate AUSMDU00074711 to pELF1-like plasmid from the same isolate. More reads align at the start or end (coloured box) of the plasmid suggesting that the plasmid is linear.

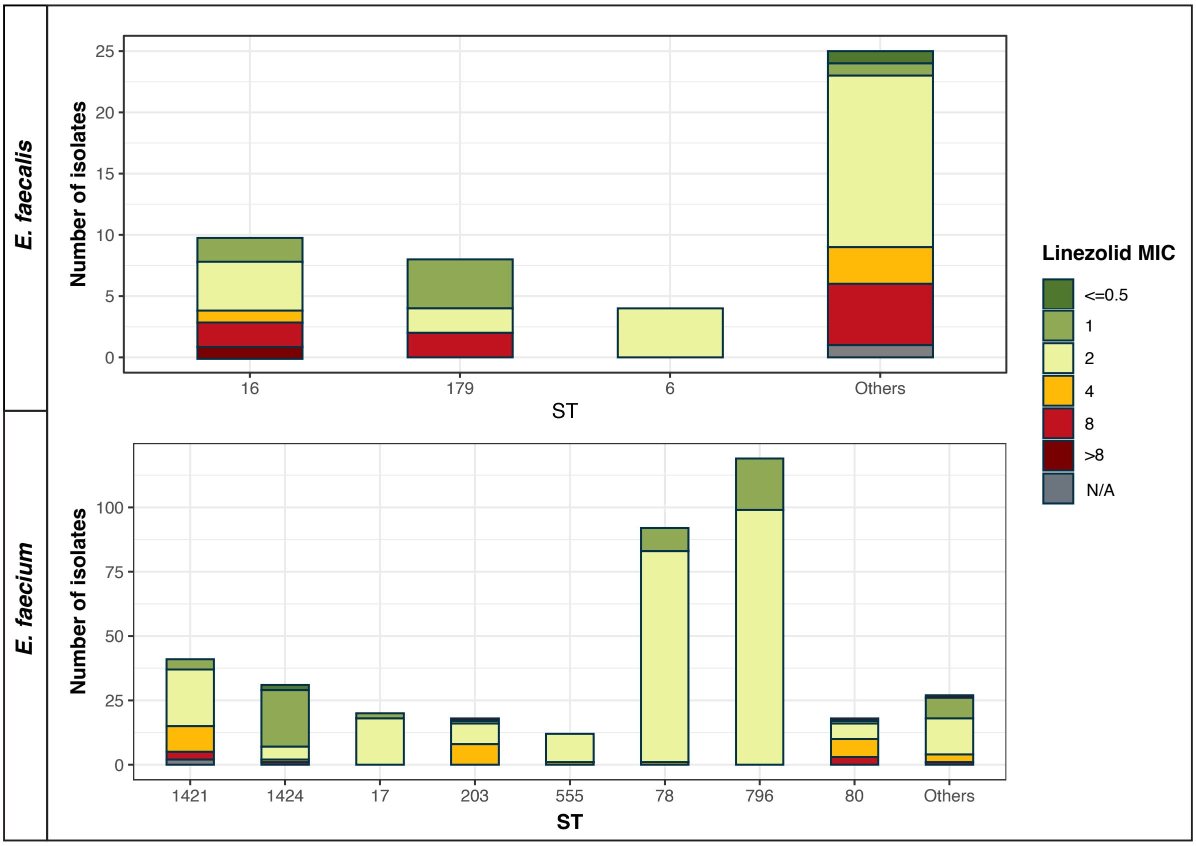

Figure S8. MLST profiles of Victorian *E. faecalis* (*N* = 47) and *E. faecium* (*N* = 379) grouped by linezolid MIC.

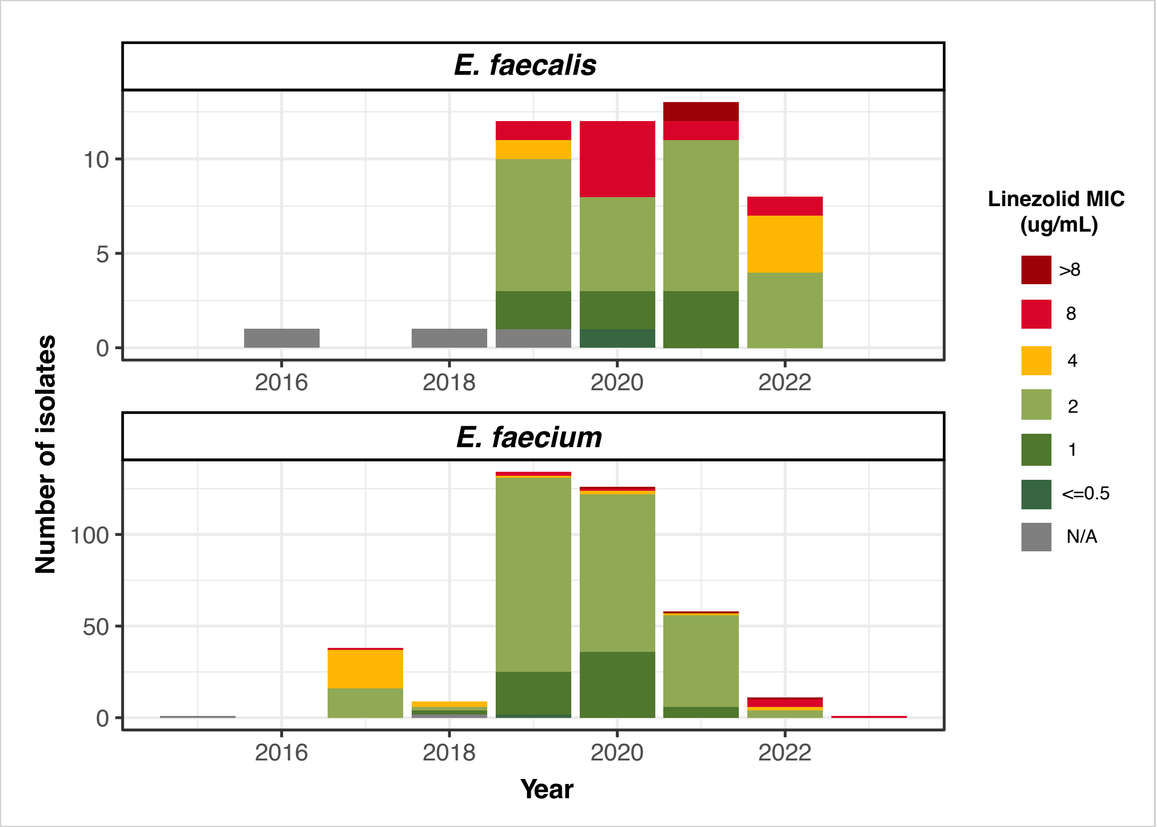

Figure S9. Linezolid MIC distribution over the years during the study period of enterococci from Victoria, Australia.

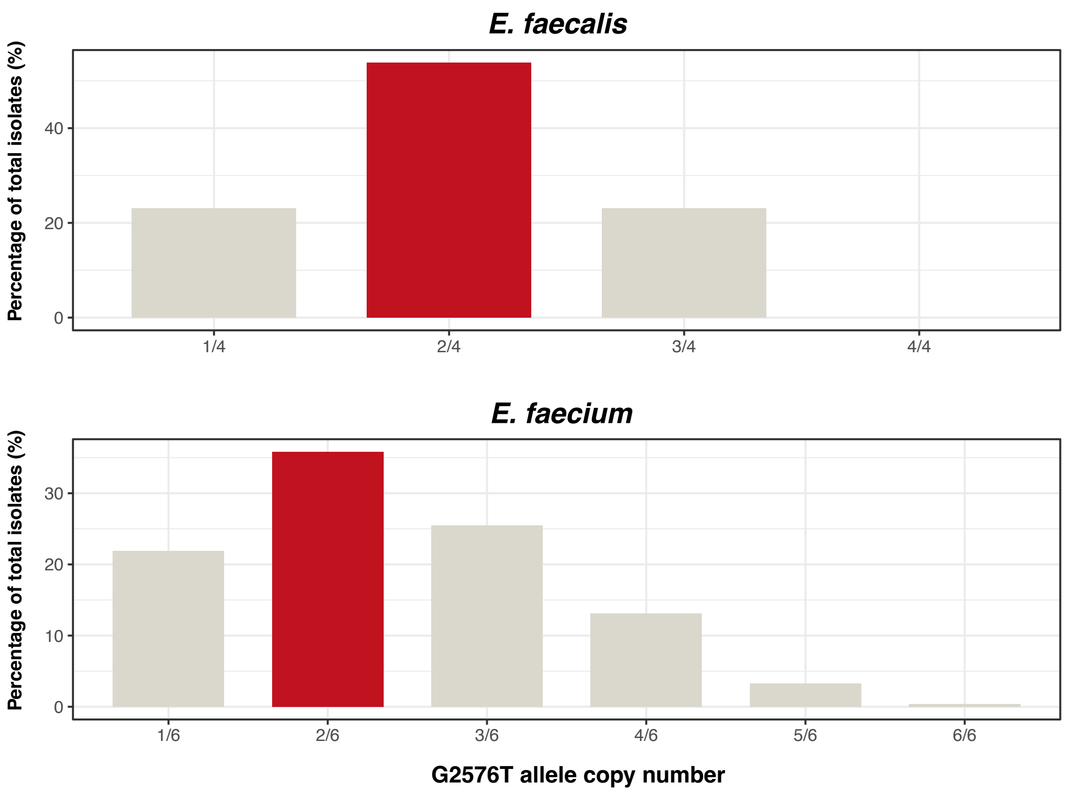

Figure S10. Copy number of 23S rRNA alleles with G2576T mutation in SRA *E. faecalis* and *E. faecium*.
